# Advancement and Challenges of Biosensing Using Field Effect Transistors

**DOI:** 10.3390/bios12080647

**Published:** 2022-08-17

**Authors:** Gokuraju Thriveni, Kaustab Ghosh

**Affiliations:** 1 Department of Electronics and Communication Engineering, School of Engineering and Technology, CHRIST (Deemed to be University), Mysore Road, Kumbalgodu, Bengaluru 560074, India; 2 Centre for Nanoelectronics and VLSI Design, Vellore Institute of Technology, Vandalur Kelambakkam Road, Chennai 600127, India; 3 Vellore Institute of Technology, School of Electronics Engineering, Vandalur Kelambakkam Road, Chennai 600119, India

**Keywords:** biosensors, field effect transistors, functionalization, screening, Debye length, sensitivity, specificity

## Abstract

Field-effect transistors (FETs) have become eminent electronic devices for biosensing applications owing to their high sensitivity, faster response and availability of advanced fabrication techniques for their production. The device physics of this sensor is now well understood due to the emergence of several numerical modelling and simulation papers over the years. The pace of advancement along with the knowhow of theoretical concepts proved to be highly effective in detecting deadly pathogens, especially the SARS-CoV-2 spike protein of the coronavirus with the onset of the (coronavirus disease of 2019) COVID-19 pandemic. However, the advancement in the sensing system is also accompanied by various hurdles that degrade the performance. In this review, we have explored all these challenges and how these are tackled with innovative approaches, techniques and device modifications that have also raised the detection sensitivity and specificity. The functional materials of the device are also structurally modified towards improving the surface area and minimizing power dissipation for developing miniaturized microarrays applicable in ultra large scale integration (ULSI) technology. Several theoretical models and simulations have also been carried out in this domain which have given a deeper insight on the electron transport mechanism in these devices and provided the direction for optimizing performance.

## 1. Introduction

Effective biosensors are an urgent need in the present age of the pandemic and health crisis. Advancement in next generation medicine and point of care (POC) diagnostics requires rapid, low cost biosensing devices with high sensitivity and selectivity. Devices based on zero dimensional nanomaterials are a popular choice in biosensing platforms such as surface plasmon resonance of gold nanoparticles towards detection of carcinoembryonic antigens in blood plasma and quantum dot-based biosensors for imaging of cancer cells [1,2,3]. However, these materials are mostly used in vivo and thus a biosensing device is required for effective detection of moieties in vitro. Amongst various biosensors developed for in vitro detection, field effect transistors (FETs) have emerged to be potentially important sensing devices due to their simple fabrication process, low cost and faster response. The device is composed of three terminals such as a source, a gate and a drain. The applied electric field at the gate is used to regulate the conductivity of the channel connecting source and drain. FET can be used in two ways for biosensing operations. In the first type of operation, charged biomolecular species bind to the channel surface and alter its conductivity due to the charge transfer process. This changes the drain current *I_D-bio_* through the channel, which is measured and compared to the current *I_D_* in the absence of the biomolecule. Alteration in this current $\left|I_{D-bio}-I_{D}\right|$ is used to detect the presence of the molecule and the extent of this alteration defines the sensitivity. In the other type of operation, the biomolecules are immobilized in the fabricated cavity adjacent to the gate dielectric layer. The presence of the biomolecule with specific dielectric constant inside this cavity alters the gate dielectric capacitance and causes a shift in threshold voltage. A change in current due to threshold voltage shift indicates the presence of the moiety.

The evolvement biosensor dates back to 1970 with the invention of ion-sensitive field-effect transistors (ISFETs), which are a combination of metal oxide semiconductors (MOS) and glass electrodes for measuring ion activities in biological and electrochemical environments [4]. Adsorption FET and hydrogen sensitive MOSFET then came into existence in quick succession [5,6]. However, all these FETs were highly bulky with more space requirements. Later in 1988, Nakamura and his team came up and devised a biosensor with a novel fabrication technique that aligned well with the complementary metal-oxide semiconductor (CMOS) fabrication process [7].

For medical diagnosis and for point of care testing, a requirement arose for a miniaturized, lightweight biosensor. The advent of nanotechnology catered to this need with the development of such miniaturized biosensing technology. The nanomaterials have unique properties such as a high surface area to volume ratio, biocompatibility, high sensitivity and good chemical stability. Hence, these are extensively used as channel materials in FET biosensors and are found to be a better replacement for conventional MOS-based technology.

A variety of novel functional nanomaterials are being used in this domain and it is important to name a few, such as ZnO nanostructures, silicon nanowire, carbon nanotubes, graphene nanoribbons and transition metal dichalcogenides. ZnO nanostructures are found to have high electron transfer rates and are non-toxic with easy preparation routes for synthesizing different shaped structures to increase the surface area to volume ratio and the sensitivity [8]. For highly sensitive and label-free detection, silicon nanowire can be used [9]. This nanomaterial also has the feasibility of large-scale manufacturing and the possibility of commercialization, albeit it has the challenges of lower carrier mobility and device-to-device variation of nanowire density and orientation. Graphene stands tall amongst the nanomaterials owing to its unique properties of outstanding tensile strength of 130 GPa and modulus of 1000 GPa, ultra-large surface area and carrier mobility that leads to ultra-fast charge transport capability, chemical and electrochemical inertness and biocompatibility [10,11]. Thus, it is extensively used in new interesting areas of research such as wearable or implantable sensor technology for development of smart, soft contact lens and in neural probes [12,13]. The innovative sensing approach in graphene also exhibits edge functionalization, that is, biomolecular conjugation at the edge of the graphene sheet for highly sensitive detection of biomolecules [14]. Nevertheless, its challenges include a zero bandgap that lowers the on-off current ratio, which is disadvantageous for several bioFET applications [15]. Thus, it is doped with different functional groups for opening the bandgap to increase sensitivity [16]. Carbon nanotubes (CNTs) share a common structural element relationship with graphene and thus exhibits higher conductivity, high mechanical strength along with excellent thermal and chemical stability [17]. The bio-probes such as enzymes and antibodies can be functionalized either on the surface or inside the hollow cavity and aid in the efficient electron transfer reactions [18,19,20]. Nevertheless, CNTs are synthesized in different shapes and chirality that cause a drastic change in electronic properties [21,22,23]. Non-carbon based 2D materials are also amongst the emerging biosensing materials with a wide range of electrical properties. Especially the high on/off current ratio exhibited by MoS_2_ makes it an attractive material for bioFET applications [24]. However, the free dangling bonds in these materials inhibit the interface charge transfer leading to Fermi energy pinning and the formation of recombination centers [25]. Two-dimensional black phosphorus (BP) nanosheets have also gained attention, as BP is the most stable allotrope in the phosphorus family with interesting electronic properties [26]. It possesses a thickness dependent bandgap varying from 0.3 eV for bulk and 2 eV for monolayers along with higher carrier mobility. All these features are reflected in its performance as a sensing channel in FET biosensors. Last but not least, the immense contribution of AlGaN/GaN high electron mobility transistors (HEMTs), which are chemically inert and thermally stable biosensors [27,28], should be mentioned. Using this device, Woo et al. successfully detected stress hormone cortisol up to the 1 pM limit of detection (LOD) [29].

The pace of advancement is also accompanied by various challenges. One such issue is the electrical screening of charges due to the electrostatic effect in a solution [30]. The distance to which this screening effect persists refers to the Debye length, which weakens the detected signal. Various molecules can be introduced to reduce this screening effect, such as aptamers and polymers [31,32]. Innovative techniques are implemented to circumvent screening such as the application of high frequency voltage waveforms and device modifications. Other than screening, it is also necessary to increase the surface area of the sensing surface for improving detection sensitivity. Along with these requirements, it is important to assess the device-to-device variation in biosensing chips, minimize power dissipation and maximize device yield. Various experimental works that have been carried out in this domain for overcoming these challenges are extensively supported by numerical modelling and simulations. These models are used to visualize the distribution of charge, charge transfer processes and potential profiles towards optimizing the parameters of bioFETs for improving performance [33,34,35]. Thus, in this review, starting from the principle of biosensing, we have provided a detailed discussion on the surface chemistry and functionalization process in bioFETs towards detection of infectious pathogens, and the critical challenges and the innovative approaches which have been implemented to tackle these hurdles. We have also explored a multiplex of theories, which have provided valuable information and insights towards bringing about required modification in these devices.

## 2. Working Principle of the Field Effect Transistor as a Biosensor

Biosensing in FETs is carried out by three operations, namely (1) biorecognition, which is the recognition of the analyte containing the biomolecular species (such as proteins, antibodies, enzymes etc.) through changes in the electronic properties of the sensor; (2) transducer operation that converts this change in the properties to an electrical signal; and (3) amplification of the signal for digital readout by the user. The first operation or the biorecognition event (*BE*) can be described mathematically by

(1) $$BE=dc\frac{d\sigma }{dc}\;\;\;\;\;\;\;\;\;\;$$

where *dc* is change in the concentration of the analyte responsible for the change in conductivity *dσ* of the sensor due to the charge transfer process. Equation (1) thus describes the response of the sensor to the presence of the analyte through changes in its electronic properties, that is, its conductivity. This is the first step towards biosensing and the sensitivity of the sensor depends on its efficacy. The mathematical equation for the next stage or the transducer (*TR*) operation is expressed by

(2) $$TR=\frac{dV_{EG}}{d\sigma }\frac{dI_{D}/dV_{EG}}{I_{D}}\;\;\;\;\;\;\;\;$$

**Figure 1:**
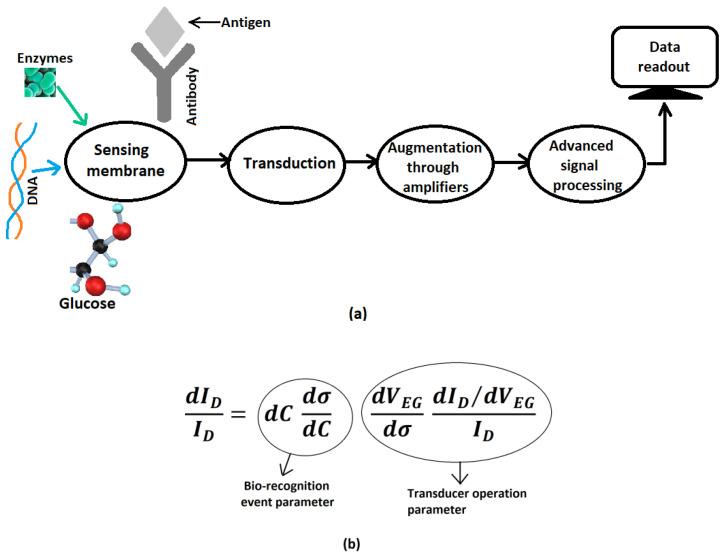
(**a**) A schematic representation of the working of a biosensor; (**b**) sensitivity formulation of biosensor depending on bio-recognition event parameter and transducer operation.

Here, the change in the conductivity alters the threshold voltage and thus the effective gate voltage *V_EG_*, which results in the variation of drain current *I_D_*. Alteration of this drain current, which is a measurable parameter, indicates the detection of the biomolecule and the extent of this alteration is related to the sensitivity. Figure 1 shows the schematic representation of the working principle of the biosensor and the formulation of the altered drain current *dI_D_* due to biorecognition event to the initial drain current *I_D_* in the absence of biosignals, which is expressed as the product of the biorecognition and transduction parameters given in Equations (1) and (2). Various types of bioFETs are fabricated and proposed that rely more on BE or on the TR parameter or on both.

**Figure 2:**
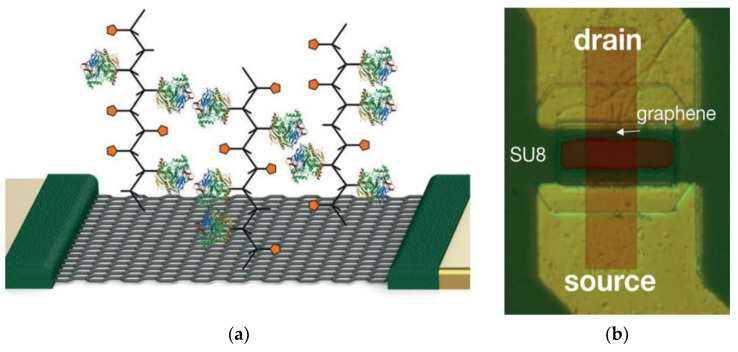
(**a**) Schematic representation of the enzyme functionalized GFET. (**b**) Micrograph of the GFET. Reprinted with permission from [36], Copyright 2014, American Chemical Society.

In some FETs, the analytes are introduced directly on the channel surface and the biomolecules are adsorbed on the surface either by chemisorption or physisorption processes as shown in Figure 2 in a graphene-based biosensor [36].

In this case, the adsorption of molecules is associated with a charge transfer process that alters the dσ/dc of the BE parameter, which is responsible for detection. For example, Dontschuk et al. experimentally demonstrated the use of graphene field effect transistors (GFETs) as probes for detecting the DNA nucleobases adsorbed on the surface [37]. Various research has also shown that the functionalization of the biomolecules at the edges of the channel surface leads to a greater change in device conductivity and sensitivity [14,16]. In other types of FETs, such as ISFETs and dielectrically modulated field effect transistors (DMFETs), the sensitivity hinges on the transconductance (*dI_D_/dV_EG_*) factor, which depends on the dielectric constant of the specific biomolecule [38,39,40]. Ohno et al. thus demonstrated linear changes in transconductance of ISFET with adsorption of protein molecules (bovine serum albumin) at low concentrations and saturation at higher concentrations [38]; Im et al. showed binding of biomolecules in DMFET that alters the dielectric constant at the gate and causes large changes in threshold voltage, which can alter the transconductance. High k gate dielectric layers thus play a vital role in these types of devices for improving sensitivity.

## 3. Functionalization of Biomolecules in FET

**Figure 3:**
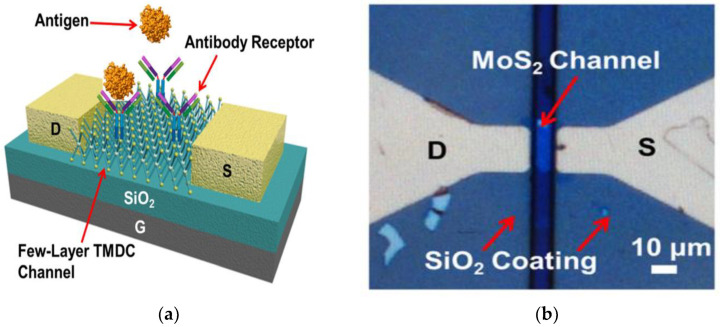
(**a**) Schematic sketch of FET biosensor with antigen-antibody binding. (**b**) Optical micrograph of MoS_2_ FET biosensor. Reproduced from [46] with the permission of AVS: Science & Technology of Materials, Interfaces and Processing.

The first important step for successful detection of proteins, enzymes and various biochemicals is the functionalization process. Both covalent and non-covalent functionalization lead to changes in the morphology of the sensing material on both local and global scales, and modification of elastic and electronic properties and transport characteristics. Covalent functionalization is the formation of covalent bonds between the surface of the sensing material and the modifier, which have a significant effect on its physiochemical properties. However, non-covalent functionalization has a lesser impact on structure and properties as compared to covalent modification and the effect is proportional to the modifier’s binding energy. To understand these modification principles and their influence on sensing mechanisms in different materials, it is required to have an in-depth knowledge on the nature and strength of interactions of the biomolecule on the sensing surface. To develop such an understanding, several theoretical models have emerged that describe the functionalization process in biosensors. These theories play a vital role in guiding the efficacy of the functionalization process through modification of the surface chemistry and the binding efficacy in nanomaterials such as silicon nanowires, graphene and its derivative and transition metal dichalcogenides. Examples include the work of Mirsian et al., who carried out a silanization reaction using (3-Aminopropyl) triethoxysilane (APTES) as initiators in silicon nanowire based sensors. Here, silanization reaction conditions of oxygen plasma, APTES concentrations, and solvent and reaction temperature are optimized for selective functionalization of silicon nanowire sensors with PSA antibodies rather than the silicon substrate surface. Guided by simulations, the approach is found to be effective in improving the sensitivity of the sensor three times more as compared to bioFET with nonselective functionalization [41]. Pykal et al. described different theoretical models and methods for simulating the functionalization process based on quantum and classical mechanics [42]. Milowska et al. presented a study on the transport properties of covalently functionalized graphene monolayer based sensors and demonstrated a mechanism to control the device sensitivity by varying the concentration, particular arrangement and type of surface groups [43]. A relevant theoretical description based on mathematical modelling with the non-equilibrium Green’s function (NEGF) method is given by Thriveni et al. for single edge and double edge functionalization and their effects on device sensitivity [16]. Supported by density functional theory, Cho et al. demonstrated for the first time that functionalization of molecules at the edges of graphene nanoribbons leads to a seven-fold improvement in sensitivity and fifteen-fold reduction in response time as compared to pristine GNR sensors [14]. All these theories along with experimental verification thus demonstrate altered electrical properties, which lead to improved sensitivity of GNR upon functionalization. Guided by this approach, innovative functionalization techniques are carried out for effective detection of chemical moieties. Bai et al. thus developed folic acid modified reduced graphene oxide for highly sensitive detection of cancer biomarkers [44]. Nasrollahpour et al. developed ultra-sensitive bioassay of HER-2 protein for diagnosis of breast cancer using reduced graphene/chitosan in a nanobiocompatible platform [45]. Apart from surface chemistry and surface interactions, the binding efficacy of the molecule on the sensing surface is also one of the important factors for biosensing. Antigen-antibody binding for detection of pathogens and other biomarkers is the most effective approaches for improving the binding efficacy of the biomolecule and diagnosing various diseases. In this method, the antibodies against specific surface proteins of the pathogen are chosen and are functionalized on the channel surface of the FET as shown in Figure 3 for a transition metal dichalcogenide FET biosensor [46]. With this technique, label-free biosensing can be achieved with higher sensitivity as well as specificity.

The first demonstration of real time pathogen (influenza A virus) detection by this technique dates back to 2004 with an antibody functionalized silicon nanowire biosensor [47]. Here, the LOD of the virus particle in the solution was 5 × 10^4^ particles/mL. Thereafter, a variety of pathogens such as bacteria and virus particles were detected, such as rotavirus, human immunodeficiency virus (HIV) and Ebola [48,49,50].

**Figure 4:**
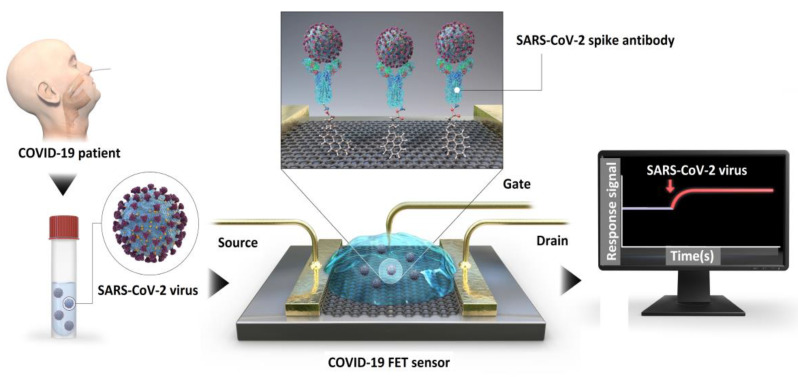
Schematic sketch of the operational procedure of graphene based COVID-19 FET sensor for detection of SARS-CoV-2 spike antibody. Reproduced from [51] with permissions granted for the duration of the World Health Organization (WHO) declaration of COVID-19 as a global pandemic.

The onset of the COVID19 pandemic led to extensive utilization of this technique for detection of SARS-CoV-2. It is noteworthy to mention the work of Seo et al., who developed a graphene field effect transistor (GFET) wherein the antibody receptor was immobilized on the surface of the graphene channel for binding with the SARS-CoV-2 spike protein as shown in Figure 4 [51]. The sensor is not only found to be highly sensitive in detecting a 1 fg/mL concentration of SARS-CoV-2 spike protein but also highly specific in not responding to a related coronavirus strain such as MERS-CoV spike protein. A similar type of detection methodology was also carried out by Cui et al. with laser induced GFET, which detected 1 pg/mL of SAR-CoV2 spike protein and also exhibited good specificity in responding only to the spike protein and no significant response to non-complementary nucleocapsid proteins [52]. Other kinds of viruses such as the Japanese encaphalitis virus (JEV) and the Avian influenza virus (AIV) are also detected efficiently by this method [53]. Here, monoclonal antibodies such as anti-JEV and anti-AIV are used for binding with the respective antigens and the limit of detection (LOD) of the viruses are found to be 1 fM and 10 fM respectively. No cross-reactivity took place for non-specific binding of the JEV antigen with the AIV antibody, exhibiting good specificity. This antigen-antibody binding is also exhibited in underlap embedded silicon nanowire field effect transistors for multiplex detection of human immunodeficiency virus (HIV) and AIV [49]. Using gp41 antibodies in rolled up InN microtubes, Song et al. was able to detect HIV with LOD of 2.5 pM in serum samples [54].

## 4. Challenges in Functionalization: Screening Effect

One important factor which cannot be ignored in the functionalization process is the net electrostatic screening effect (also known as the Debye screening effect). The Debye screening length is calculated by the formula

(3) $$\lambda_{D}=\frac{\varepsilon kT}{q\sqrt{C}}\;\;$$

where *C* is the ionic strength, *ε* is the dielectric constant, *T* is the temperature in absolute scale of the biological sample solution and *k* is the Boltzmann’s constant. For a normal physiological solution, the Debye length is about 0.7 nm, which is much smaller than protein molecules such as antigens and longer length antibodies around 12 nm. With such larger lengths, an electrical double layer is formed that screens the net charges of electrons within the antigen antibody binding zone and causes fundamental problems in detection. 

Reducing the strength of the ionic solution, that is, by a desalination process or by limiting the volume available for ions to form a double layer can be used to extend the Debye length and reduce screening. Thus, for effectively detecting proteins, electrical measurements are conducted in diluted buffer solution in 0.1× or 0.01× PBS, where the Debye length can be increased to 2.4 and 7.4 nm respectively. Chen et al. thus desalinated the biological test solution by filtering salt ions with a blood dialyzer, which was effective in detecting serum tumor markers using double gate silicon nanowire transistors [55]. Another way to reduce the strength of the solution is by deprotonation and increasing the pH level of the solution for controlling the Debye length, as carried out by Vacic et al. [56]. Kang et al. also showed that the capacitance change due to variation in pH in the interface between the solution and the sensing electrode needs to be considered for detecting protein molecules in nanoFET biosensors [57]. However, diluting the ionic strength of the solution causes alterations in protein structure and loss of protein activity as well as binding efficacy.

## 5. Effective Strategies to Overcome Screening Effect

### 5.1. Using Solution with High Dielectric Constant

Increasing the dielectric constant of the solution can also be used to overcome the screening limitation. Here, porous and biomolecular permeable polymers can substantially change dielectric properties in aqueous solutions and increase the Debye screening length for detecting biomolecules in high ionic strength solutions [58]. Gao et al. thus used a biomolecular permeable polymer layer for increasing the effective screening length in the region immediately adjacent to the GFET sensor, which led to selective detection of cancer markers [59].

### 5.2. Fragmentation Technique

**Figure 5:**
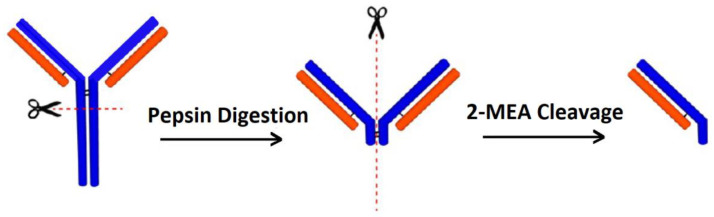
Schematic sketch illustrating the fragmentation of antibody molecule. Enzyme pepsin is used to cut the lower portion of the antibody. 2-mercaptoethylamine (2-MEA) is used as a reducing agent for further fragmentation. Reprinted with permission from [60], Copyright © 2012, American Chemical Society.

In this process, only the antigen binding part of the antibody is fragmented as shown in Figure 5 to reduce the size of the receptor so that the biorecognition event occurs at a closer proximity to the sensor surface. Using a nanowire-based FET device and involving this fragmentation technique, successful detection of proteins down to a sub-pM concentration range could be realized in untreated serum and blood samples [60].

### 5.3. Usage of Aptamers

**Figure 6:**
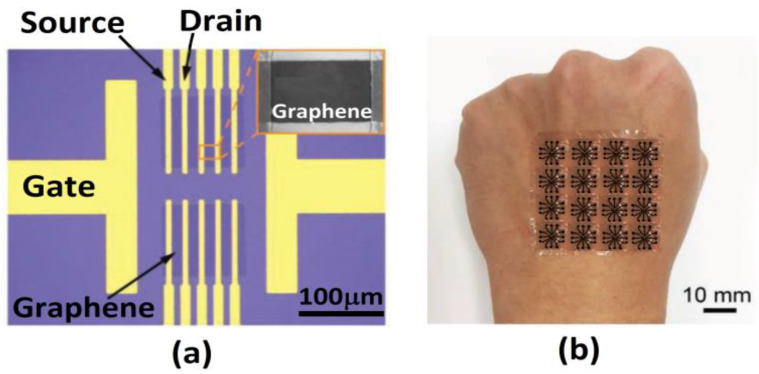
(**a**) Image of aptamer-based graphene nanosensor with on-chip source, drain and gate electrodes. Inset shows SEM image of electrodes on the graphene conducting channel. (**b**) Photographs of the sensor mounted on human hand. Reprinted/adapted with permission from [65], Copyright 2019, John Wiley and Sons-Books.

Instead of longer length antibodies, aptamers can be used, which are short strands of oligonucleotides with length ranging from 10–60 bp. Binding of the target molecules with aptamers can occur within the electrical double layer even in 20–50 mM salt solution. Thus, aptamer-based FET biosensors exhibit robust and selective detection of target biomolecules in undiluted biological samples such as serum, blood and brain tissues for usage as implantable neural probes. It also has high detection sensitivity. Nakatsuka thus detected glucose molecules in the 10 pM–10 nM concentration range under high ionic strengths by using deoxyribonucleotide aptamers in ultra-thin metal oxide field effect transistor arrays and Farrow et al. designed a thin film transistor functionalized with aptamers for spike protein detection of COVID-19 within the 1 pM to 1 nM range [31,61]. Due to its chemical simplicity, rapidity and enhanced stability, aptamers can also be utilized in environmental monitoring towards detection of various contaminants and toxic targets that might prove difficult to detect with antibodies. Thus, aptamer biosensors or aptasensors are used to monitor water quality by detection of arsenic, heavy metals such as lead, cadmium and mercury, pesticides, bacterial toxins and other industrial byproducts [62]. Likewise, these sensors are also incorporated in various food and agricultural products as well for determining food safety through detection of various mycotoxins in food such as ochratoxin A, aflatoxins, Fumonisin B1 and Zearalenone [63]. The inherent qualities of aptamers also open up the possibility for incorporation in wearable sensors for improving sensitivity, selectivity and biocompatibility along with decreasing production costs. Here, the probe conjugated aptasensor with DNase present in the skin surface hydrolyzes DNA, which causes rapid, sensitive and specific detection of biomolecules [64]. A graphene conducting channel modified with an aptamer on ultra-flexible thin film of Mylar is thus successfully incorporated in a skin patch and in contact lens as shown in Figure 6 [65]. Selective aptamers are also used in wearable plant sensors for usage in agriculture and crop management [66].

### 5.4. Usage of Nanobodies

Nanobodies fall among the shortest biological receptors with lengths lesser than 3 nm, which facilitate analyte binding closer to the sensor surface. These are much smaller and structurally simpler than antibodies (15 nm) and even antibody fragments (7–8 nm), can also be easily produced and have remarkable physicochemical stability under varied conditions. Thus, nanobody receptors are used in carbon nanotube transistors for highly sensitive, selective and label free-protein detection in physiological solutions [67]. It can also be used as a tool for diagnosis and treatment of cancers, and detection of environmental pollutants and food borne microbes [68,69].

### 5.5. High Frequency Electronics in Nanobiosensing

**Figure 7:**
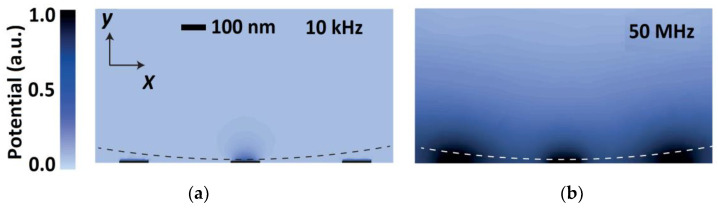
Spatial distribution of AC potential in 10 mM salt solution at (**a**) low modulation frequency of 10 kHz and (**b**) high modulation frequency of 50 MHz. Adapted with permission from [73], Copyright 2015 Springer Nature.

Besides using aptamers or fragmenting the antibodies as described, time varying voltage waveforms can be applied to the FET for overcoming screening limitations. If the frequency of the waveform is low and less than 1 MHz, there is sufficient time for the ions in the biomolecular solution to form an electric double layer (EDL) and screen the charges. A further increase in frequency weakens the formation of the EDL as the ions fails to settle back in equilibrium, which impedes the screening. This method is highly effective, and several studies have been carried out in this field. For modelling the formation of EDL in the presence of ionic concentration gradients and fluid flow, Poisson’s equation is coupled with the Nernst-Planck equation to develop a new Poisson-Nernst-Planck (PNP) system of equations [70,71,72]. The equations describe the electronic environment surrounding the electrodes and analytes modulated by an externally applied electric field. Laborde et al. thus performed a three-dimensional finite-element simulation based on the PNP formalism in the frequency domain [73]. Figure 7 shows the map of AC potential amplitude in 10 mM salt solution at a modulation frequency of 10 kHz and 50 MHz [73]. Here, the dashed line shows the position of the microsphere formed by the dielectric layer. The simulation portrays the penetration of electric field only up to a few Debye screening lengths into the solution when operated at a lower modulation frequency of 10 kHz, which extends deep into the solution with an increase in modulation frequency to 50 MHz. Supported by simulations, the researchers also developed a CMOS nanocapacitor array. They were able to carry out real-time imaging of microparticles and cancer cells in physiological salt solutions using high frequency impedance spectroscopy with high spatial and temporal resolutions.

**Figure 8:**
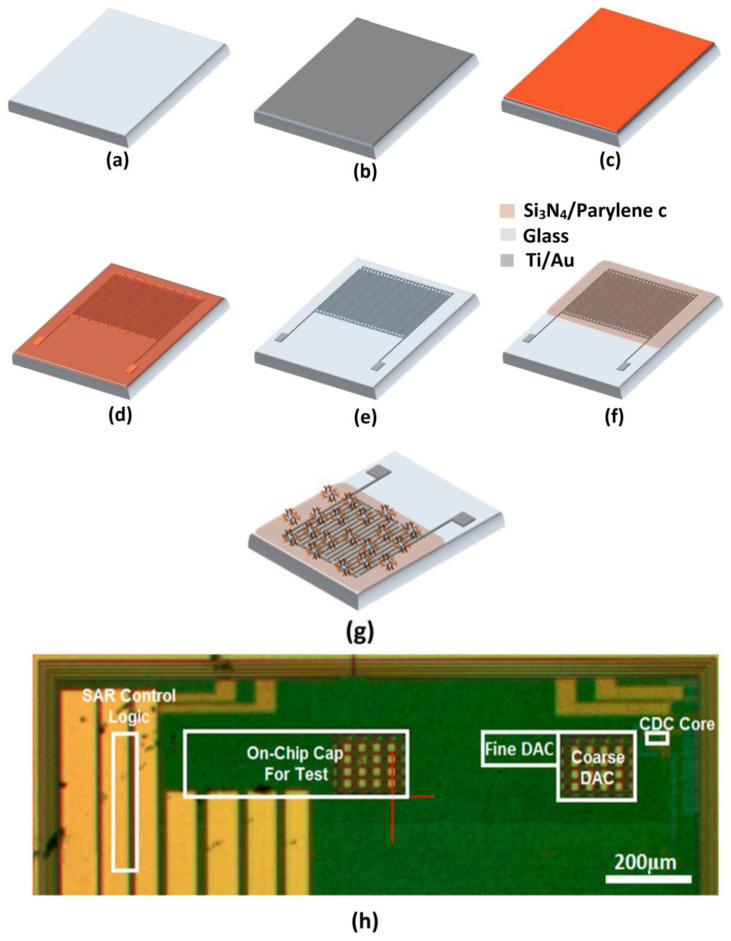
Fabrication procedure of CMOS capacitive biosensor using photolithography: (**a**) cleaned glass substrate; (**b**) sputtering deposition of electrode terminals such as Ti and Au; (**c**) coating of photoresistor; (**d**) patterning of the resistor; (**e**) after photoresistor development, dry etching and removal of the resistor; (**f**) dielectric deposition and realization of the capacitive sensor array with interdigitated electrodes; (**g**) immobilized antibodies on the sensor surface; (**h**) packaged sensor chip mounted on the printed circuit board. Reproduced from [74] under the Creative Commons Attribution License.

Figure 8 shows the fabrication process of a CMOS capacitive biosensor using a photolithography technique towards the development of a packaged chip. It also depicts the immobilization of antibodies on the surface of the sensor [74]. Widdershoven et al. measured the impedance spectroscopy with this type of CMOS pixelated nanocapacitive biosensor for detecting polymeric microspheres and living cells. The microspheres are deposited on the top of the pixel and the performances of the sensor are analyzed at various frequencies. Screening is found to occur at low frequencies and the spheres could be sensed by the pixels close to the surface of the chip. The screening effect is reduced as the frequency is increased up to 50 MHz and spheres can also be detected by adjacent pixels as the electric field from the electrode extends beyond the Debye length. The method can be utilized to discern the spheres with different dielectric properties and to image different kinds of cells moving across the pixels [75,76].

Cossettini et al. further used the PNP equations to describe the virus particles where they compared the capacitance signal of a cowpea chlorotic mottle virus with just the capsid of the same virus [77]. They found that a low frequency signal is more sensitive to analyte charges in the nanoelectrode, whereas a high frequency signal is sensitive to analyte volume. A finite element method simulator is also developed by Liu et al. to solve the PNP equations for describing the transport of the charged molecules within synthetic nanopores [78,79]. The study illustrates that electro-diffusion current flow in electrolyte solutions significantly suppresses the screening of the biological charge and leads to ten-fold amplification of the signal.

**Figure 9:**
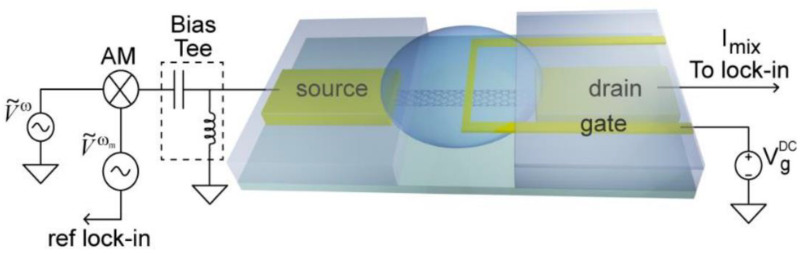
Nanoelectronic based heterodyne sensor. Reprinted with permission from [80], copyright 2012, American Chemical Society.

A different technique is used by Kulkarni et al. using a nanoelectronic heterodyne sensor, shown in Figure 9 [80]. Here, they used single walled carbon nanotubes and applied nonlinear mixing between the AC excitation field and the biomolecular dipole field. This generates mixing currents sensitive to surface-bound molecules. They were able to detect monolayer streptavidin binding to biotin in 100 mM buffer solution at high frequency signals beyond 1 MHz.

### 5.6. Device Modifications

**Figure 10:**
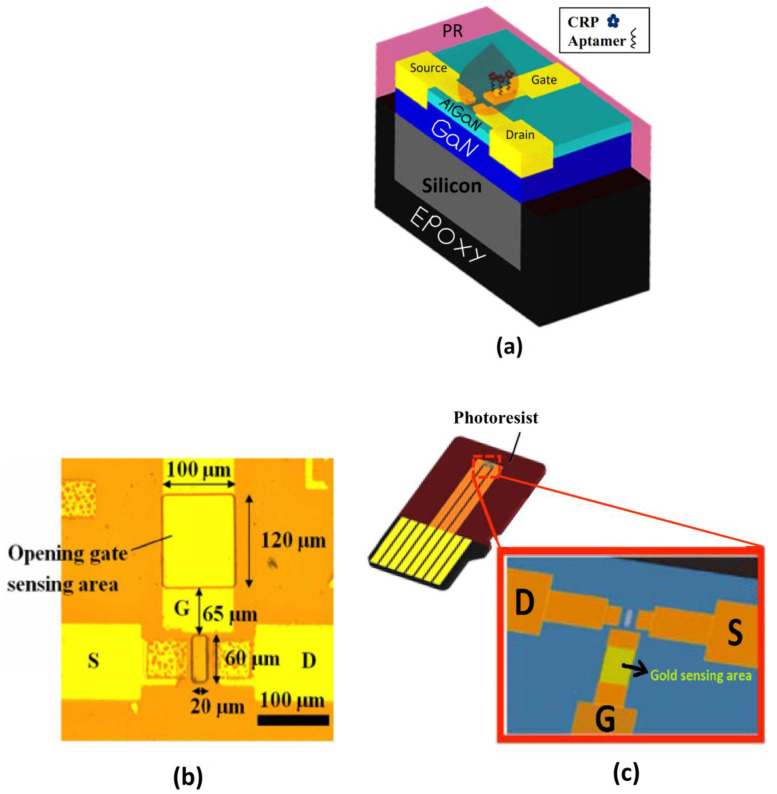
(**a**) Schematic sketch of GaN/AlGaN HEMT. (**b**) Top view of the device showing sensing region consisting of channel opening (20 × 60 μm^2^) and extended gate electrode opening (100 × 120 μm^2^); the distance between the transistor channel and gate electrode is 65 μm. (**c**) Showcasing of the device from the fabricated chip. Reproduced from [28] under the terms of Creative Commons Attribution Non-Commercial No Derivatives 4.0 License.

Various modifications in device technology have emerged to combat screening limitations for efficient detection of biosignals. One such device is a miniaturized AlGaN/GaN high electron mobility transistor embedded in a plastic substrate and connected with metal electrodes. The device can be used to directly detect the biomolecules in high ionic strength solution, even in 1× PBS or human serum, without compromising sensitivity [27,28]. In this device, a narrow microchannel is fabricated. Through this channel, the test liquid is driven by the capillary effect to deliver in the sensing region as shown in Figure 10. Here, an extended gate is used for separating the channel from the chemical and biological environment. The device is driven by the biomolecular solution capacitance rather than the conventional charge-based detection process in FETs and hence the charge screening issue is circumvented. Application of gate voltage and bias voltage at the source drains terminals and causes a voltage drop Δ*V_S_* across the solution, forming an electrical double layer in the interface between the extended gate and the channel. This constitutes the solution capacitance *C_S_*, which modulates the drain current through the channel. We find the following relation between gate voltage *V_G_*, Δ*V_S_* and voltage drop Δ*V_dl_* across the dielectric layer of the FET.

(4) $$V_{G}=\Delta V_{S}+\Delta V_{dl}\;\;$$

(5) $$\Delta V_{dl}=\frac{\frac{1}{j\omega C_{dl}}}{\frac{1}{j\omega C_{dl}}+\frac{1}{j\omega C_{S}}}V_{G}=\frac{C_{S}}{C_{dl}+C_{S}}V_{G\;\;\;\;\;\;\;\;\;\;\;\;}$$

where ω is the angular frequency and *C_dl_* is the dielectric layer capacitance. The value of *C_S_* increases with increase in ionic strength of the solution, which leads to larger effective *V_G_* on the dielectric layer of the FET, causing a larger increase in drain current. Thus, higher current gain can be achieved in a solution with higher ionic strength.

**Figure 11:**
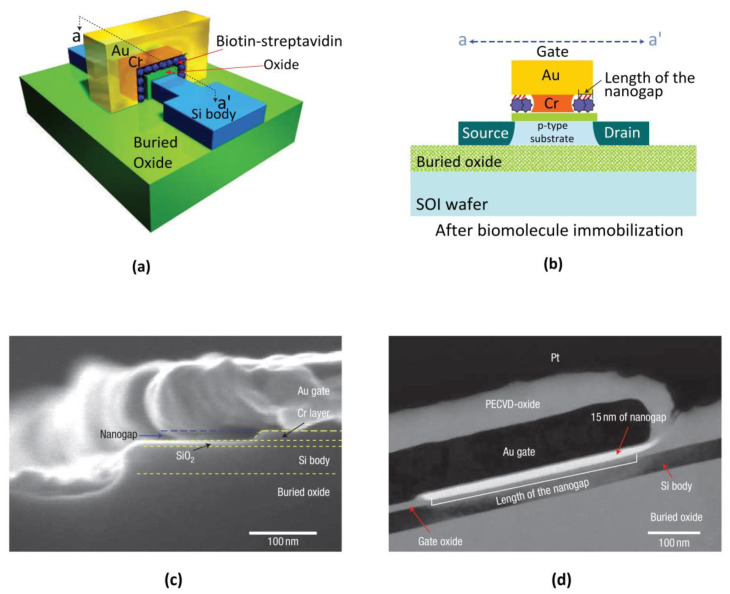
Schematic sketch of the (**a**) 3D structure; (**b**) 2D structure of DMFET exhibiting the air gap that can be filled by biomolecules; (**c**) SEM image and (**d**) TEM image of the nanogap cavity in the DMFET. Reprinted with permission from [40], Copyright 2007 Springer Nature.

The same kind of concept can be applied for a dielectric modulated field effect transistor (DMFET) biosensor. Here, a vertical nanogap is fabricated near to the edge of the gate dielectric using thin film deposition and wet etching as carried out by Im et al. [40] and shown in Figure 11. Depending on the dielectric constant of the biomolecule, the total capacitance of the DMFET can be significantly altered, leading to a shift in the threshold voltage. The DMFET biosensor thus fabricated by Im et al. is highly sensitive to the specific binding of streptavidin to biotin, which caused a large shift in the threshold voltage. Gu et al. also fabricated a label-free biosensor with nanogap embedded FET. They found a remarkable change in the transistor parameters as the gate dielectric constant is changed by filling the nanogap with biomolecules and detected specific binding between the avian influenza antibody with antigen of silica binding protein [81]. Kim et al. also fabricated DMFET for label free detection of DNA [82]. They found a shift in the threshold voltage to the negative side for neutralized DNA and to the positive side for negatively charged DNA. They further fabricated a nanogap embedded CMOS (NeCMOS) and found that the threshold voltage shift is four times greater in a p-channel device than in an n-channel one, and a decrease in the nanogap length enhances the sensitivity [83].

Various theoretical models have also interpreted the performance characteristics of DMFET. Kannan et al. proposed a dielectric modulated impact ionization MOS (DIMOS) transistor for label-free biomolecule detection with a TCAD simulation study, which indicated high sensitivity to the presence of biomolecules even at small channel lengths [84]. Rahman et al. developed a compact I-V model of a monolayer MoS_2_-channel-based DMFET for detection of biomolecules in dry environments [85]. Using a SILVACO ATLAS 2D Device simulator, Singh et al. proposed a dielectrically engineered Schottky barrier MOSFET for operation in overlapped biosensing mode [86].

## 6. Challenges with Surface Area and Effective Solutions and Strategies

Enhancing the specific surface area of the sensor is one of the important factors for improving the sensitivity. Catering to this need, three-dimensional graphene foam having porous hollow structure is synthesized for biosensing applications. Compared to the two-dimensional graphene sheet, graphene foam not only restores intrinsic properties of graphene but also possesses high compressing strength with extremely large surface area. Xu et al. thus devised a graphene foam field effect transistor (GF-FET) for detecting adenosine triphosphate (ATP) down to the 0.5 pM level. Following the same technology, Song et al. also developed a three-dimensional GFET (3D-GFET) for detecting microRNA from the 100 pM to 100 nM range [87].

Other effective techniques include the usage of nanoribbon structures rather than nanowires. This increases the surface area for detection and boosts up the sensitivity. Ma et al. thus fabricated a dual gate silicon nanoribbon-based ion sensitive field effect transistor for direct label-free detection of protein molecules in high ionic strength solution [88] and also Cordyceps Sinensis with a LOD of 10 and 50 pM respectively [89].

**Figure 12:**
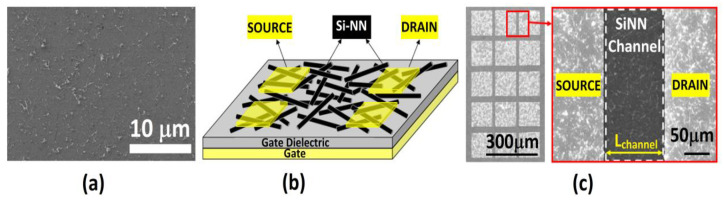
(**a**) SEM image of SiNN (**b**) Si-NN FET structure and (**c**) SEM image of fabricated SiNN FET. Reproduced from [90] under the Creative Commons Attribution License.

To introduce reproducibility in electrical properties from one nanowire device to another, a new transistor technology was developed with randomly deposited silicon nanowire networks (SiNN), called silicon nanonet field effect transistors as shown in Figure 12 [90]. The nanostructure networks not only have high specific surface area but are also tolerant to defects. Nyugen et al. successfully demonstrated, for the first time, integration of SiNN into a low cost sensor for label free DNA detection [90,91].

Based on numerical simulation, a very important insight on surface to volume ratio dependent sensitivity was put forth by Shoorideh et al. Using an analytical argument, they showed that electrostatic screening is weaker near to the concave surface and stronger at the convex side. Thus, detection sensitivity is enhanced in concave corners of the sensing materials due to reduced screening effect, and they concluded that larger surface area to volume ratio is not responsible for enhancing the sensitivity [92]. They also optimized the biasing point of the sensor that maximizes sensitivity and also identified Debye-Hückel screening near the oxide/electrode interface that is responsible for bias dependent charge induction in the sensor [93]. The theory proposed by Shoorideh et al. is validated in deformed graphene channel field effect biosensors for ultrasensitive detection of nucleic acids [94]. Researchers found that compared to a flat graphene surface, the counter-ions in the concave region of deformed graphene are distributed over a longer distance away from the surface of graphene. This resulted in decreased screening of DNA molecules at the concave surface, which validates the proposed theory. 

## 7. Challenges in Device Yield and Device-to-Device Variation

Ultimately, the miniaturized biosensor microarrays and microfluidic systems are integrated in a lab-on a chip system, called a biochip. Major criteria which need to be assessed in these chips are the percentage of device yield and device-to-device variation. Both yield and the device-to-device variations are due to formation of different types of defects during the fabrication process such as synthesis, surface chemistry, pattern transfer, etching, lift-off, deposition of contacts during metallization etc. In a biosensing chip, device yield refers to the percentage of devices that are working properly within the specified limits and tolerance windows and are not damaged during the fabrication process. Device yield thus signifies the quality of the fabrication process and the maturity of the integrated device. Wang et al. thus obtained more than 98% device yield for wafer scale fabrication of separated carbon nanotube thin film transistors and the few unconductive devices are due to peel-off of metal contacts during the fabrication steps [95]. Arunyadet et al. reported 100% device yield for In_2_O_3_ nanoribbon biosensors using the simple 2 mask lithography process [96].

Apart from having high device yield, it is also required to have smaller device-to-device variation for improved performance in biosensing. This variation refers to the deviation in the electrical performance of the individual sensors in the array or chip. This poses a serious obstacle for biosensing applications as prior calibration is required for each of these devices. Need for calibration increases the chance of user errors, leading to incorrect response of the sensor. Thus, device-to-device variation needs to be suppressed, which requires rigorous simulations and calculations for guiding innovative fabrication techniques. Honda et al. thus fabricated a practical impediment biosensor having parallel plate electrodes (PPE) with an insulated electrode edge that enables current to be uniformly distributed on a planar electrode surface [97]. This improved the device-to-device variation as compared to interdigitated microelectrode (IDE) arrays. However, before fabrication, they simulated the current density of the electrodes and found that the electric current is highly concentrated on the edge corner of the IDE and thinly distributed on the flat surface. From this simulation data, they were able to fabricate the PPE structure to improve the reproducibility of the sensor. Ishikawa et al. demonstrated a calibration method in nanowire biosensors in which the ratio of the absolute response or change in current (ΔI) to the gate dependence on conductance (dI_DS_/dV_G_) for different devices in the array behaves almost identically [98]. This helped them to substantially suppress the device-to-device variation, allowing the usage of the sensors in large arrays. Tu et al. thus fabricated a graphene FET array biosensor (6 × 6 GFETs on chip) for detection of mercury ions with an ssDNA aptamer. In mixed solutions containing various metal ions, the device showed outstanding selectivity to Hg^2+^. The detection limit was fairly low, below 40 pM, and the device showed a wide range of detection from 100 pM to 100 nM and faster response below 1 s [99].

## 8. Challenges in Current Leakage, Power Dissipation and Proposed Solutions

**Figure 13:**
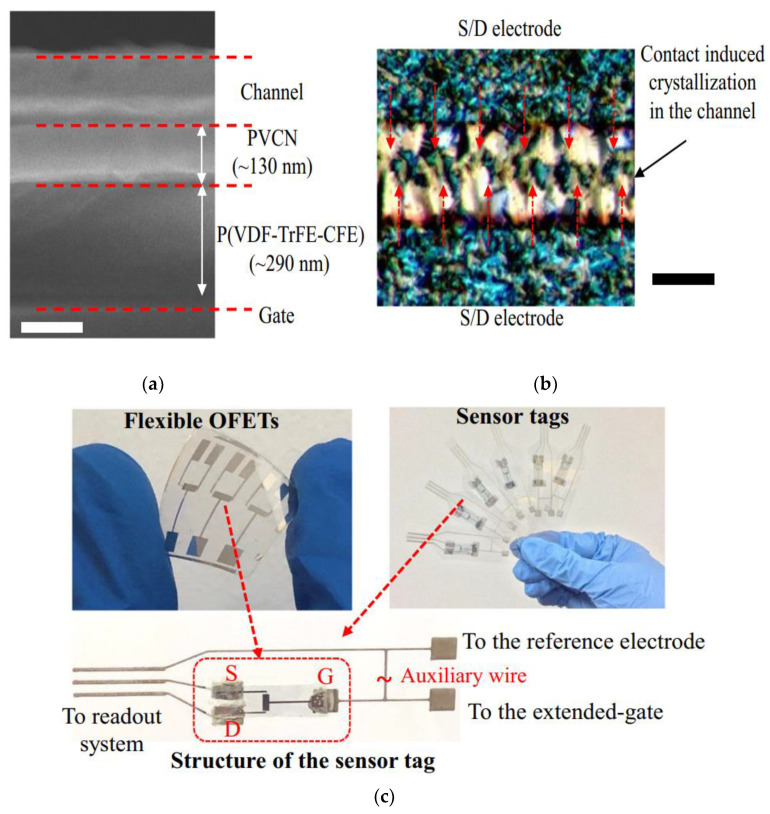
(**a**) Cross-sectional SEM image exhibiting the material stacking of OFET; (**b**) polarized optical micrograph of the OFET depicting the contact-induced crystallization in the channel with perfluorobenzenethiol-modified silver source and drain electrodes; (**c**) fabricated OFETs and the sensor tag images depicting the extended-gate sensing and reference electrode. Reproduced from [101] under the Creative Commons Attribution 4.0 International License.

Miniaturization of devices to nanoscales enables incorporation of more than one million transistors in a single chip, which is referred to as ultra-large scale integration (ULSI) technology. Ever-increasing demand for high speed operation in these ULSI chips requires high clock frequencies. This increases the power dissipation and the operating temperature of the chip. Device reliability issues thus arise due to electro-migration and hot carrier device degradation. Thermal stress can also cause heat dissipation, which is also another major reliability concern. To counter this effect, cooling systems are implemented to keep the temperature in the acceptable range, which ultimately overshoots the manufacturing cost. Thus, the biosensing device used in the chip needs to be designed effectively for minimizing current leakage and power dissipation. Organic field effect transistors (OFETs) cater to this need for low power consumption along with environmental compatibility, which also meet the requirement of flexible devices for on-body wearable sensors and medical monitoring equipment. Bhatt et al. thus developed fully flexible electrolytic gated field effect transistors on flexible polyethylene terephthalate substrates for pH sensing and for detection of prostate specific antigens operating at a lower voltage range from 1–1.5 Volts [100]. Tang et al. fabricated low power organic field effect transistors (OFETs) as shown in Figure 13 for label free detection of miRNA, a potential biomarker for primary breast cancer, with high sensitivity and specificity at a lower operation voltage < 1 Volts [101].

Apart from flexible electronics, fabrication of low power devices also involves device modifications such as in dielectrically modulated L shaped gate tunneling field effect transistors (DM-LTFETs), which are suitable for high sensitivity and low power consumption biosensors [102]. Further development of low power biosensors requires a thorough investigation on various computational models used to minimize power without compromising on performance. Towards this end, Thriveni et al. and Damodaran et al. provided a detailed description and modelling of various types of leakage and power dissipation in a MOSFET and quantum dot-based modulation doped field effect transistor (MODFET), respectively [103,104,105]. The findings exhibited the need for a high k dielectric layer with larger conduction band offset (CBO) or a high band gap semiconductor layer for reducing leakage and minimizing power consumption. Jana et al. formulated the current sensitivity, delay and power consumption in a dual cavity junction less double gate MOSFET-based biosensor and also investigated the impact of charged biomolecules on the aforesaid parameters [106].

## 9. Future Directions and Conclusions

This review introduces the advancement of biosensing devices starting from the late 1970s to present-day innovations in low power ULSI technology. Amongst these various sensing devices, field effect transistors are found to be of immense interest due to lower cost, faster response and simple fabrication processes. Thus, over the years, the transistor technology has transformed from bulky ISFETs to nanoscale FETs. Functionalization of biomolecules on the FET surface is now well understood with various theoretical models, which led to effective binding of the molecules through antigen-antibody interactions and surface chemistry modifications. The methodology proved to be highly effective in the recent coronavirus outbreak, which has witnessed extensive usage of FET based sensors towards RT-PCR and serological tests of the masses. This is due to the efficiency of the device towards detecting the SARS-CoV-2 spike protein of the virus with high sensitivity and selectivity. However, the pace of advancement is also accompanied by some critical issues. In this review, we have thoroughly discussed those issues and the innovative approaches undertaken to find a solution, without compromising the performance. One such challenge is the Debye screening effect, which screens the electrical charges in antigen-antibody binding interactions and hampers the detection. The solution lies in increasing the Debye length so that the screening does not take place at the site of the biorecognition event. Decreasing the strength of the ionic solution can be carried out to increase the Debye length and overcome screening. However, this degrades binding efficacy of the molecules. Other effective strategies which can be implemented to reduce screening are listed here:

- Fragmentation method in which antigen binding part of the long length antibody is fragmented for the biosensing event to occur closer to the sensor surface and evade the screening effect: This can be used to detect protein molecules down to sub-pM concentration ranges;
- Using short chain aptamers rather than antibodies for detecting the targeted molecules from the 1 pM to 1 nM range;
- Increasing the dielectric constant of the ionic solution for detecting prostate specific antigen at 1 nM concentration.

Screening can also be overcome by preventing the formation of an electrostatic double layer rather than increasing the Debye length or by utilizing the biomolecular solution capacitance rather than the charge transfer process. The methodologies are again listed here:

➢ Application of high frequency voltage waveforms in FET: The method is found to be useful in detecting biomolecules even in high ionic strength solutions. Applying this strategy, two important biosensing devices emerged such as the CMOS pixelated nanocapacitive biosensor and the nanoelectronic heterodyne sensor;
➢ Using an AlGaN/GaN HEMT device with microchannel capillaries to drive the biomolecular solution to the sensing region: Here, the disadvantage of screening is turned to an advantage as the electrical double layer forms the solution capacitance that controls the current through the channel. A higher ionic strength solution thus enhances the capacitance, which increases the sensitivity and current gain of the device;
➢ Using a DMFET with vertical nanogap near to the edge of the gate dielectric: Here, the sensitivity of the device depends on the dielectric constant of the biomolecules and thus the detection process is not hampered by the charge screening effect.

Enhancing the surface area of the sensor is also another important factor which can boost the sensitivity of the sensor. Based on this, several theoretical interpretations are given which illustrate the physical concepts and insights on the charge screening mechanism in the convex/concave interface of the device. Supported by these models, the sensors are modified accordingly, and a few important device structures are described here:

❖ Three-dimensional graphene foam field effect transistor with porous hollow structure and extremely large surface area;
❖ High surface area nanoribbons rather than nanowire, such as a silicon nanowire field effect transistor for detecting biomolecules in high ionic strength solution;
❖ Silicon nanowire networks with extremely high specific surface area and tolerant to defect generation.

In the current world of lightweight devices implemented in the internet of things (IoT) based ecosystem, it is imperative to look forward towards integrating the microarray devices and micro-fluidic channels in the form of biochips. High device yield and smaller device-to-device variation are some of the primary requirements for the fabrication of these chips. The chips also need to sustain high clock frequencies and at such high frequencies it is crucial to analyze the device reliability. This needs effective design of FETs for minimizing current leakage and power consumption. Here, two types of transistors can be used, which are listed here:

▪ Organic field effect transistors can be used for low power operation with operating voltage < 1 Volts;
▪ Dielectrically modulated transistors can also be used to minimize current leakage and power consumption. Here, a high k dielectric layer needs to be chosen with higher conduction band offset over the bandgap of the semiconductor channel.

The knowhow of all the innovative approaches and techniques as discussed in this review can provide a direction towards the fabrication of low cost, miniaturized biosensing devices and chips that not only offer higher detection sensitivity and selectivity but also exhibit better reliability to high frequency signals in low power ULSI technology.

## Data Availability

Not applicable.
